# Progress in the Use of Echocardiography in Patients with Tumors

**DOI:** 10.31083/j.rcm2501022

**Published:** 2024-01-15

**Authors:** Tiantian Feng, Zhaoxia Guo, Hongling Su, Fu Zhang, Hai Zhu, Aqian Wang, Kaiyu Jiang, Bo Li

**Affiliations:** ^1^The First Clinical Medical College of Gansu University of Chinese Medicine (Gansu Provincial Hospital), 730000 Lanzhou, Gansu, China; ^2^Department of Cardiology, Pulmonary Vascular Disease Center (PVDC), Gansu Provincial Hospital, 730000 Lanzhou, Gansu, China

**Keywords:** echocardiography, tumor patient, cardiovascular disease, cardiac structure and function

## Abstract

Advances in cancer treatment have increased patient survival rates, shifting clinical focus towards minimizing treatment-related morbidity, including cardiovascular issues. Since echocardiography allows for a comprehensive non-invasive assessment at all cancer stages, it is well suited to monitor cardiovascular disease secondary to oncology treatment. This has earned it significant attention in the study of cardiac tumors and treatment-induced cardiac alterations. Ultrasound methods—ranging from transthoracic and transesophageal echocardiography to ultrasound diagnostic techniques including myocardial strain imaging, myocardial work indices, three-dimensional cardiac imaging—offer a holistic view of both the tumor and its treatment impact cardiac function. Stress echocardiography, myocardial contrast echocardiography, and myocardial acoustic angiography further augment this capability. Together, these echocardiographic techniques provide clinicians with early detection opportunities for cardiac damage, enabling timely interventions. As such, echocardiography continues to be instrumental in monitoring and managing the cardiovascular health of oncology patients, complementing efforts to optimize their overall treatment and survival outcomes.

## 1. Introduction

Cardiovascular disease (CVD) often co-occur with tumors, an effect attributable to both innate tumor development and the effects of anticancer therapy [1]. In fact, up to 36% of tumor patients experience cardiovascular diseases, arising from a combination of direct tumor effects, therapy, and the accumulation of risk factors [1]. These patients face elevated risk of cardiovascular death, stroke, heart failure, and pulmonary embolism [2]. Notably, heart failure and pulmonary embolism risks become more pronounced several years after tumor diagnosis [2]. Patients with genitourinary, hematological, neurological tumors, and lung cancer exhibit the highest cardiovascular risk [2].

The 2022 European Society of Cardiology (ESC) Guidelines on cardio-oncology advocate for baseline risk assessment to identify high risk oncology patients [3]. For those assessments and follow-ups, echocardiography is the preferred cardiac imaging method [3]. It offers non-invasive, real-time, and reproducible data for early detection of cardiac issues in oncology patients [3]. A summary of echocardiography use in patient care is presented in Table 1.

**Table 1.** **Guidelines related to oncologic heart disease**.

NO	Year	Title	Consensus
1	2022	2022 European Society of Cardiology (ESC) Guidelines on cardio-oncology developed in collaboration with the European Hematology Association (EHA), the European Society for Therapeutic Radiology and Oncology (ESTRO) and the International Cardio-Oncology Society (IC-OS): Developed by the task force on cardio-oncology of the ESC	Before cancer treatment initiation, physicians should identify and treat cardiovascular (CV) risk factors (CVRF), pre-existing CVDs, and define an appropriate prevention and surveillance plan for early identification. After completing cancer treatment, the focus shifts to coordination of long-term follow-up and treatment.
2	2022	Cardio-Oncology Recommendations for Pediatric Oncology Patients	The use of novel therapies has expanded the focus of cardiotoxicity timing of from delayed effects to acute and delayed toxicity. Closer monitoring and earlier surveillance is required.
3	2020	Management of cardiac disease in cancer patients throughout oncological treatment: ESMO consensus recommendations	Concerns about anticancer therapy CV damage should be weighed against the potential benefits of cancer therapy.
4	2019	Cardio-Oncology: Vascular and Metabolic Perspectives A Scientific Statement From the American Heart Association	Vascular complications in patients with cancer represent a new challenge for the clinician and a new frontier for research and investigation.

ESMO, European Society for Medical Oncology; CVDs, cardiovascular diseases.

## 2. Echocardiography for the Evaluation of Patients with Tumors

### 2.1 Evaluation of Cardiac Function and Structure

#### 2.1.1 Left Ventricular Systolic Function

While oncology treatments have significantly increased cancer cure rates in recent years, they also pose a substantial cardiotoxicity risk, elevating mortality rates [4]. Therapy must balance oncologic efficacy with cardiac safety [3]. Specifically, a 2-year mortality rate as high as 60% has been observed in patients who develop heart failure (HF) due to oncology treatments [4]. Up to 26% of breast cancer and leukemia/lymphoma survivors experience cardiotoxicity or overt HF even decades after recovery [5]. The leading drugs associated with oncologic treatment-induced cardiotoxicity include anthracyclines and human epidermal growth factor receptor 2 (HER2) inhibitors [6]. Other contributing agents encompass alkylating agents, antimetabolites, antimicrotubular medications, monoclonal antibodies (e.g., trastuzumab), as well as interferon and bleomycin [6]. Given these risks, early detection and intervention for cardiotoxicity is critical [1].

The American Society of Echocardiography (ASE) and European Association of Cardiovascular Imaging (EACVI) define oncology treatment-induced cardiotoxicity as a decrease in left ventricular ejection fraction (LVEF) of more than 10%, but less than 53% [7]. This diagnosis is confirmed by subsequent cardiac imaging 2–3 weeks after the baseline study [7]. If patients’ LVEF remains within the normal range, they are not considered to have cardiotoxicity and continue with their treatment [7]. The 2022 American Heart Association (AHA)/American College of Cardiology (ACC)/Heart Failure Society of America (HFSA) guidelines outline four key purposes for pre-treatment LVEF assessment in oncology patients: (1) pre-treatment risk stratification and diagnosis of pre-existing cardiomyopathy, (2) establishing a baseline for future comparative re-evaluation, (3) initiating cardioprotective medications before cancer treatment, and (4) guiding the choice of cancer therapies [8]. The most common technique for measuring LVEF is the two-dimensional (2D) Simpson biplane method. However, its reliability can waver when the endocardium is obscured, often due to pulmonary air interference [9]. Alternatively, three-dimensional (3D) LVEF measurement offers advantages, such as reduced inter-observer variability [10] and a strong correlation with cardiac magnetic resonance (CMR) imaging. Despite its precision, 3D LVEF measurement remains highly examiner dependent [11]. While CMR may serve as the gold standard for LVEF measurements when echocardiographic quality is suboptimal, it is limited by the need for repeated scans [12]. Emerging techniques including speckle tracking imaging, longitudinal strain imaging, and stratified strain of the myocardium offer more accurate LVEF assessment during antineoplastic therapy (Table 2, Ref. [9, 11, 12, 13]).

**Table 2.** **Clinical studies and consensus of echocardiography on left ventricular systolic function**.

NO	First author	Year	Main findings
1	Lang RM [9]	2015	The two-dimensional Simpson method is the most common method for measuring LVEF.
2	Thavendiranathan P [13]	2014	Reduced variability in each measurement when measuring ventricular function in three-dimensional compared to two-dimensional.
3	Lambert J [11]	2020	Three-dimensional -derived LVEF correlates well with cardiac CMR and provides high accuracy.
4	Esmaeilzadeh M [12]	2022	CMR as the gold standard for measuring LVEF.

LVEF, left ventricular systolic function; CMR, cardiac magnetic resonance.

#### 2.1.2 Left Ventricular Diastolic Function

The main indicators of Diastolic function include mitral diastolic flow velocity (E, A), mitral E peak deceleration time (DT), left ventricular lateral and septal motion velocity (e’), Mitral Doppler (E/e’) [14]. In recent years, several studies have demonstrated that radiotherapy drugs can induce ventricular diastolic insufficiency, with over 47% of patients showing heart failure even with stable LVEF [15]. For instance, Armstrong *et al*. [16] used echocardiography to evaluate treatment-related cardiac insufficiency in 1820 adult survivors of childhood cancer who received anthracycline-based chemotherapy. Their findings indicate that abnormalities in cardiac diastolic function were more prevalent than those identified through 3D-measured LVEF [16]. Results from the St. Jude Lifetime Cohort Study (SJLIFE) suggest that relying solely on LVEF measurements may not provide a comprehensive picture of cardiac function, particularly in adult survivors of childhood cancer [16]. Thus, long-term follow-up and monitoring of diastolic function using echocardiography allows early detection of adverse cardiac events in oncology patients.

Supporting this notion, Upshaw *et al*. [17] early stages of antineoplastic therapy were associated with abnormal left ventricular diastolic function (LVDF) often manifested before any changes in systolic function. The study also highlighted the relative sensitivity of the E/e’ parameter [17]. Similarly, Lu Cao *et al*. [18] confirmed these finding and further identified a link with concurrent radiotherapy. In this context, Calabrese *et al*. [19] demonstrated that serial LVEF measurements were not consistently effective in detecting patients at high risk for tumor-induced cardiotoxicity. Their study revealed that within just a week of starting anthracycline therapy, 36% of patients were diagnosed with asymptomatic diastolic dysfunction, despite having preserved LVEF levels [19]. These patients were monitored using echocardiography for cardiac function assessment [19]. Suggests that ventricular diastolic dysfunction occurs with relatively normal LVEF in some of the treated tumor patients. In conclusion, ventricular diastolic dysfunction is not negligible in patients undergoing oncologic therapy, early monitoring of left ventricular diastolic function for cardiotoxicity in oncologic patients provides greater sensitivity than LVEF, and it is useful to document baseline diastolic function for general cardiac risk assessment and for follow-up during cancer therapy (Table 3, Ref. [16, 17, 18, 19]).

**Table 3.** **Major clinical studies of left ventricular diastolic dysfunction after echocardiography assessment of antineoplastic therapy**.

NO	First author	Year	Sample num	Main findings
1	Upshaw JN [17]	2020	362 patients treated for breast cancer	Antineoplastic therapy leads to an earlier Ventricular diastolic function deterioration compared to systolic function.
2	Cao L [18]	2015	40 radiotherapy combined/32 adjuvant radiotherapy/71 cases of radiotherapy alone	LVDF is more likely to detect cardiotoxicity early compared to LVEF.
3	Armstrong GT [16]	2015	1820 adult survivors of childhood-onset cancer	One-third of survivors with normal three dimensional-LVEF have evidence of underlying diastolic dysfunction. Survivor screening assessment should include a comprehensive diastolic function assessment.
4	Calabrese V [19]	2018	80 chemotherapy patients with LVEF >50% and normal diastolic function	Asymptomatic diastolic dysfunction with preserved LVEF was diagnosed in 36% of patients examined 1 week after chemotherapy.

LVEF, left ventricular systolic function; LVDF, left ventricular diastolic function.

#### 2.1.3 Right Ventricular Function

During both pre- and post-chemotherapy phases, it’s crucial to evaluate right ventricular function and pulmonary artery pressure through echocardiographic monitoring in oncology patients [20]. Chemotherapy can induce structural changes to both the right and left ventricles. The impact on the right ventricle, in particular, should not to be overlooked. The major chemotherapeutic agents that have a significant effect on the right ventricle include: anthracyclines which induce irreversible cardiomyocyte death by targeting topoisomerase II β, trastuzumab causes dilated cardiomyopathy by inhibiting tyrosine kinase receptor 2, cyclophosphamide leads to pulmonary hypertension due to oxidative stress-induced endothelial damage, and dasatinib which alters the endothelial to pulmonary artery smooth muscle cell ratio, skewing it towards anti-proliferation resulting in pulmonary hypertension [21]. Commonly, a peak systolic tricuspid regurgitation (TR) velocity >2.8 m/s is indicative of a moderate likelihood of pulmonary hypertension [22]. Moreover, specific markers signal right ventricular dysfunction and carry prognostic implications for the progression of heart failure. These include a tricuspid annular plane systolic excursion (TAPSE) <17 mm, a fractional area change (FAC) <35%, and Tissue Doppler tricuspid annular systolic velocities (S’) <9 cm/s [22].

Echocardiography serves a dual function during oncology treatment: the assessment of right ventricular structure and function—which may deteriorate either concurrently with or prior to left ventricular dysfunction—and supports the early cardiotoxicity detection [23]. Research by Abdar Esfahani *et al*. [24] indicates that TAPSE and FAC are useful in assessing the right ventricular function, that Tissue Doppler is a sensitive tool to assess chemotherapy-induced right ventricle damage even when LVEF readings are normal, and that some antineoplastic therapeutic agents can induce pulmonary hypertension. However, another study suggests that while antineoplastic therapy can impair both left and right ventricular function, a decline of right ventricular function doesn’t necessarily correlate with left ventricular cardiotoxicity [25]. Given the low sensitivity of conventional echocardiographic parameters in detecting right ventricular impairment due to oncologic treatment, a more comprehensive approach—incorporating strain measurements, CMR, or radionuclide angiography—is advisable for a more accurate assessment of right ventricular ejection fraction (Table 4, Ref. [24, 25]).

**Table 4.** **Major clinical studies of echocardiography to assess right ventricular dysfunction after antineoplastic therapy**.

NO	First author	Year	Sample num	Main findings
1	Abdar Esfahani M [24]	2017	67 patients treated for breast cancer	Anthracyclines may disproportionally affect the right ventricle vs. the left, even with normal LVEF. Certain antineoplastic drugs may induce pulmonary hypertension.
2	Mazzutti G [25]	2021	25 patients with breast cancer treated with TTZ	TTZ treatment leads to decreased right ventricle function without the associated left ventricular cardiotoxicity.

LVEF, left ventricular systolic function; TTZ, trastuzumab.

#### 2.1.4 Assessment of the Left Atrium

While left ventricular systolic dysfunction is the most commonly monitored form of cardiotoxicity during cancer therapy, it often manifests late in the course of treatment [26]. Recent studies suggest that left atrial enlargement may serve as an early indicator of cardiotoxicity [27]. Specifically, the left atrial index has emerged as an independent predictor of left ventricular dysfunction following cancer treatment, with high values correlating with a greater the probability of cardiotoxicity [27]. Impaired left atrial systolic function appears most frequently in breast cancer Treatments, and is strongly influenced by age [28]. As such, older patients require more stringent monitoring to facilitate timely treatment adjustments [28]. A study by Hyukjin Park and colleagues [29] used echocardiography to assess left ventricular global longitudinal strain (LVGLS) and peak amplitude longitudinal strain (PALS) in patients who underwent chemotherapy without additional treatment for cardiac dysfunction. The study found that declines in PALS were more sensitive and specific than declines in LVGLS for predicting cardiac dysfunction [29]. Thus, continuous PALS measurements could offer a more reliable parameter for predicting future cardiotoxicity [29]. In summary, evaluating left atrial structure and function adds a valuable dimension to cardiotoxicity risk assessment during cancer therapy (Table 5, Ref. [27, 28, 29]).

**Table 5.** **Major clinical studies of echocardiographic assessment of the left atrium after tumor treatment**.

NO	First author	Year	Sample num	Main findings
1	Bergamini C [27]	2018	162 patients treated against breast cancer	Left atrial index may be an independent predictor of cardiotoxicity.
2	Timóteo AT [28]	2019	100 breast cancer patients treated with chemotherapy	During follow-up, left atrial systolic function was significantly reduced in 20.8% of patients, with age emerging as the only independent predictor.
3	Park H [29]	2020	72 breast cancer patients without CTRCD after chemotherapy and additional trastuzumab treatment	After chemotherapy, PALS decline happened before significant CTRCD and without echocardiography abnormalities.

CTRCD, cancer treatment–related cardiac dysfunction; PALS, peak amplitude longitudinal strain.

#### 2.1.5 Heart Structure

Oncology treatment can induce significant cardiac structural changes, including myocardial damage and ventricular remodeling [30]. Echocardiography serves as the preferred diagnostic method for such evaluations [31]. Early phases of antineoplastic therapy may result in localized or widespread myocardial thickening and thinning, accompanied by reduced myocardial motion and alterations in ventricular chamber size [32]. These changes can lead to subsequent valvular regurgitation [32]. Additionally, Chest Radiation Therapy has the potential to cause direct valvular damage and aggravate ventricular remodeling [32]. In advanced cancer cases, patients are at risk for developing thrombotic pulmonary hypertension, which is due to thrombosis and other non-bacterial redundant embolism stemming from coagulation dysfunction and tumor cell embolization in small pulmonary vessels [33]. Activation of the coagulation system contributes to fibrous endothelial gifting of small pulmonary arteries and eventually structural changes in the heart [33]. For more in-depth assessment, an esophageal echocardiogram is recommended, with the use of CMR if the echocardiographic results are inconclusive.

### 2.2 Evaluation of Valvular Lesions Induced by Radiotherapy and Chemotherapy

While chemotherapeutic agents typically do not directly impact heart valves, they can contribute to valve damage through secondary infectious endocarditis or thrombus formation [34]. Common chemotherapeutic agents including anthracyclines, alkylating agents, and tyrosine kinase inhibitors adversely affect arterial elasticity and can induce aortic calcification during and after treatment [35]. Therefore, it’s crucial to evaluate aortic elasticity before and after cancer therapy to implement protective measures against aortic and valvular complications [35]. Long-term monitoring with echocardiography is recommended [35]. Chemotherapy increases infection susceptibility due to immunosuppression state caused by a reduced blood cell counts [36]. Lowered neutrophil levels facilitate bacterial colonization on heart valves, leading to thrombus formation, especially in patients with pre-existing valve disease [36]. Moreover, the prolonged use of chemotherapy drugs via implanted intravenous catheters or central venous lines in cancer patients increases the risk of bloodstream infection [36]. The best method for detecting infective endocarditis is transesophageal echocardiography. However, it requires skilled operation to ensure accurate and safe results. Ultrasonographers should remain vigilant for valvular and arterial lesions in patients undergoing chemotherapy to minimize complications.

Patients exposed to radiation therapy are at risk of developing radiologic valvular heart disease (RIVHD), a condition arising from osteogenic transformations in valve interstitial cells that lead to calcification. Research by Nadlonek *et al*. [37] found that radiation increased the expression of osteogenic factors in human aortic valve mesenchymal cells such as bone morphogenetic protein 2, bone bridging protein, alkaline phosphatase, and runt-related transcription factor-2 (Runx2). The predominance of valvular lesions on the heart’s left side may be related the closer radiation zone proximity to the radiation zone [38]. Furthermore, there was a higher prevalence in patients receiving radiotherapy compared to patients receiving other treatments [38]. Radiation-associated heart disease is often managed through cardiothoracic surgery [39]. However, the thickening of the junction between the base of the anterior mitral valve leaflet and the aortic root elevates surgical mortality rates [39]. Wethal *et al*. [40] observed a significantly higher incidence of valvular disease 22 years after radiotherapy compared to 10 years.Valvular complications generally manifest several years after radiotherapy and escalate with longer treatment durations. Further, the risk of developing valve disease is dose-dependent, intensifying at cumulative doses exceeding 30 Gray [41]. In conclusion, patients treated with radiation therapy should undergo lifelong echocardiographic monitoring for the early detection of RIVHD enabling timely intervention and mortality reduction (Table 6, Ref. [37, 38, 40, 41]).

**Table 6.** **Major clinical studies of echocardiography evaluation of valve lesions due to post-radiotherapy**.

NO	First author	Year	Sample num	Main finding
1	Nadlonek NA [37]	2012	Human aortic valve interstitial cells irradiated with cesium (n = 4)	Radiation-induced osteogenic phenotype in human aortic valve mesenchymal cells.
2	Bijl JM [38]	2016	50 HL survivors treated with MRT/32 HL survivors not treated with MRT	Valvular disease prevalence rises over time after radiation in HL patients treated with MRT.
3	Wethal T [40]	2009	Echocardiographic monitoring conducted 10–20 years after radiotherapy in 116 HL cases	Heart valve disease that worsens progressively following RT requires lifetime monitoring.
4	Cutter DJ [41]	2015	80 VHD patients/200 healthy controls	The incidence of heart valve disease rises with higher RT doses.

HL, Hodgkin’s lymphoma; MRT, mediastinal radiotherapy; VHD, valvular heart disease; RT, radiotherapy.

### 2.3 Evaluation of Tumor-Induced Pericardial Disease

#### 2.3.1 Pericardial Effusion and Cardiac Tamponade

Malignant pericardial effusion is most frequently observed in lung cancer, breast cancer, leukemia, lymphoma, and malignant melanoma [42]. This can occur at any cancer stage, making routine evaluation essential [42]. According to the 2015 ESC guidelines, ultrasound is the preferred diagnostic tool for pericardial disease and can categorize effusions by size: small (<10 mm), medium (10–20 mm), and large (>20 mm) [9]. It is crucial to differentiate these from epicardial fat, which is more echogenic and displaced with the myocardium during the cardiac cycle [43]. Quick accumulation of 150–200 mL pericardial fluid of pericardial can result in life-threatening cardiac tamponade [44]. In contrast, larger, slowly accumulating effusions may be better tolerated [44]. The main echocardiogram features include diastolic right ventricular free wall, systolic right atrial collapse, paradoxical septal motion, cardiac oscillation in the pericardial sac, elevated flow in the tricuspid and pulmonary valves while reducing flow in the mitral, and aortic valves during inspiration [45]. If transthoracic echocardiography is inconclusive, transesophageal echocardiography (TEE) can offer a more precise evaluation [44]. If the etiology of pericardial effusion is clear, thoracic intrapericardial therapy can be performed by diagnostic pericardiocentesis with catheter implantation [46]. Echocardiography-guided percutaneous puncture ensures a safer drainage process with a minimal risk of cardiac puncture [46]. This method also aids in determining the most appropriate surgical approach [46].

#### 2.3.2 Constrictive Pericarditis

In oncology patients, echocardiography serves as the primary imaging modality for assessing recurrent pericarditis resulting from radiotherapy [46]. The underlying mechanism of constriction in radiation-induced pericarditis involves an initial phase of microvascular damage, followed by the accumulation of fibrin and exudate, which are ultimately replaced by collagen-producing, leading to pericardial fibrosis and constrictive pericarditis (CP) [42]. Characteristic echocardiographic signs of CP, a condition marked by elevated ventricular diastolic pressure, include pericardial thickening, pronounced ventricular septum diastolic rebound, E/A values >2, significant inspiratory variation in mitral E wave velocity (>25%), widening of the inferior vena cava, and reversed hepatic venous expiratory diastolic flow [47]. Studies have found a higher respiratory variability in the mitral E region for constrictive pericarditis (mean 30.7%) compared to milder variations observed in restrictive cardiomyopathy and severe tricuspid regurgitation groups (mean 13.7%) [48]. Consequently, changes in mitral inflow velocities or deceleration times can serve as useful indicators to differentiate CP from restrictive cardiomyopathy and tricuspid regurgitation; these should be monitored over multiple cardiac cycles for a prolonged period of time. Tissue Doppler imaging reveals the medial mitral annulus velocity may be higher than the lateral annulus [45]. If the echocardiographic results are inconclusive, supplementary imaging techniques such as magnetic resonance imaging (MRI) may be employed for further clarification.

### 2.4 Evaluation of Cardiac Tumors

In cancer patients, the probability of intracardiac tumors or thrombi development is elevated. Consequently, echocardiography should focus on identifying cardiac masses, as their detection can substantially alter treatment strategies and prognostic outlook. These masses must be clearly differentiated from thrombi, non-bacterial vegetations, and pericardial cysts. Developing a clear distinction between the cardiac space-occupying lesion and the surrounding tissue is essential for differentiating between benign and malignant tumors, multi-view ultrasound settings can further refine this assessment [49]. A preferred method for detecting cardiac tumors is TEE, providing high resolution tumor visualization to determine tumor location, size, attachment, and mobility, typically with a measurement accuracy of 1–3 mm [50]. This may be complemented by cardiac computed tomography (CT) and MRI for a comprehensive diagnosis [50]. Lei Liu *et al*. [51] reviewed the clinical data of patients with space-occupying cardiac lesions in the past 10 years and analyzes their echocardiographic features, pathological diagnosis, and prognosis. The accuracy of transthoracic echocardiography (TTE) in correctly diagnosing the lesion is 91 percent [51]. The results suggested that echocardiography is a valuable tool for characterizing space-occupying cardiac lesions. It can provide important preoperative diagnostic information for cardiothoracic surgeons [51].

#### 2.4.1 Primary Cardiac Tumors

Cardiac tumors account for 0.2% of all tumors [52]. They can be classified as primary or secondary, with secondary being 20–40 times more prevalent [52]. Primary cardiac tumors are relatively rare with 75% being benign. Mucinous neoplasms are the most common with approximately 75% originating in the left atrium [53]. The atrial septum is the most common site of attachment and has a heterogeneous appearance on ultrasound cardiograms with a thin stalk attached to the septum [53]. Cardiac papillary fibroelastoma is the second most common primary cardiac tumor in adults [54]. It usually originates in the middle of the valve, mostly in the left valve, with the aortic valve being the most common, and is characterized by a small speckled tumor tip that is evident on echocardiography [54].

Nearly 25% of primary cardiac tumors are malignant [54], with Cardiac Hemangiosarcoma being the most prevalent among them [55]. This malignancy predominantly affects the right heart and frequently invades the atrioventricular wall and valves [55]. Therefore, the echocardiographer should carefully observe patients with tumors over multiple cardiac cycles for the presence of attachments in each atrioventricular cavity, and identify individual characteristics of each mass to differentiate between tumor types. Despite the value of imaging methods such as echocardiography for cardiac tumors, histology remains the best method for diagnosing tumors and developing definitive or palliative treatment plans.

#### 2.4.2 Secondary Cardiac Tumors

Secondary tumor invasion to the heart can occur through four primary mechanisms. (i) Direct invasion: malignant tumors occurring in tissues and organs adjacent to the heart (lung cancer, breast cancer, mediastinal lymphoma, etc.,) produce pericardial effusion that spreads directly through the fascia and can be shown as intrapericardial masses, which are mostly fixed and immobile with lobular appearance and non-uniform echogenic density during TTE examination [56]. (ii) Hematogenous dissemination: cancer cells enter the heart via the bloodstream, as seen in melanoma, lymphoma, and sarcoma [57]. Melanoma has been reported to occur in both the right and left heart [57]. Although cardiac metastases are rarely diagnosed pre-mortem, they can be detected using a multimodal approach, and patients with melanoma should have a careful echocardiogram performed [57]. (iii) Lymphatic metastasis: tumor cells invade lymph nodes or mediastinal lymph nodes to reach the heart, represented by lung cancer which can involve the pericardium and epicardium [56]. (iv) Venous metastasis: the typical cases include renal cell carcinoma, hepatocellular carcinoma, uterine smooth muscle tumor and pheochromocytoma. In patients with low venous blood pressure, mass obstruction can lead to blood stasis and subsequent thrombosis, often forming at the top of the tumor [56]. For instance, hepatocellular carcinoma metastasizes to the right atrium via the inferior vena cava, frequently accompanied by thrombosis [58]. Given these complexities, sonographers should meticulously evaluate the inferior vena cava and pulmonary veins during echocardiographic examination [58]. By carefully tracking the mass’s origin from the right atrium to the inferior vena cava across multiple cardiac cycles, early cardiac metastases are less likely to go undetected [58]. In light of these challenges, advancements in ultrasound technology, such as 3D echocardiography and myocardial ultrasonography, offer enhanced precision in characterizing cardiac masses and furnish valuable insights for the subsequent treatment of patients with tumors.

### 2.5 New Ultrasound Technologies

#### 2.5.1 Strain Imaging

Myocardial strain imaging quantifies myocardial deformation along longitudinal, circumferential, and radial axes [59]. Using speckle tracking technology (STE), this approach offers several advantages over Tissue Doppler methods: it is angle-independent, less influenced by loading conditions, and provides a thorough analysis of myocardial mechanics [59]. It is particularly useful in monitoring cardiotoxicity due to radiotherapy, with global longitudinal strain (GLS) serving as the most accurate measurement [59]. Several studies have shown changes in myocardial deformation precede LVEF changes, that myocardial injury occurs from the onset of radiotherapy, and that overall radial and axial myocardial strain values are abnormal in survivors with advanced tumors, even when LVEF is normal [60]. This early 10%–15% decrease in GLS can be the most useful parameter in predicting cardiotoxicity [13]. At this point, a follow-up echocardiogram is essential, and referral to an oncologist for additional treatment is mandatory. Although 3D strain techniques offer more comprehensive data acquisition compared to two-dimensional (2D) methods, they are less user friendly [61]. It has been reported in the literature that the stratified strain technique, developed from Two-dimensional speckle tracking echocardiography (2D-STE), provides accurate assessments of the overall and segmental velocity, displacement, strain, and strain rate across various cardiac layers—endocardium, mesocardium, and epicardium [62]. This layer-by-layer tracking of acoustic speckles enhances the precision of measuring cardiac functional impairment and offers more targeted clinical treatment guidance [62]. In conclusion, to identify treatment-induced cardiotoxicity in cancer patients at an earlier and more accurate stage, relying exclusively on conventional LVEF may postpone the diagnosis. Myocardial strain imaging allows for a more timely and accurate diagnosis of cardiac dysfunction. Hence, strain imaging should be extensively utilized to monitor cardiac function, thereby enriching clinical decision-making.

#### 2.5.2 New Techniques for Myocardial Work

Emphasizing early detection and management of cardiac dysfunction is crucial in cancer patients receiving potentially cardiotoxic chemotherapy. Although several studies have confirmed the high value of GLS in monitoring cardiotoxicity, it is susceptible to blood pressure fluctuations [63]. Myocardial work (MW) can serve as an adjustment parameter for these variations calculated using concepts derived from the pressure-volume loop (PVL) and the pressure-length loop [63, 64]. Myocardial work originated from the concepts of the left ventricular PVL and the pressure-length loop [64]. Suga *et al*. [64] proposed PVL in 1979, describing the correlation between pressure and volume changes in the left ventricle during a cardiac cycle. PVL is capable of assessing cardiac function and reserve capacity in physiologic and various pathologic states [64]. Calvillo-Argüelles *et al*. [65] found that in breast cancer patients receiving potentially cardiotoxic chemotherapeutic agents with lower systolic blood pressures (16–18 mmHg), a lower myocardial work index (MWI) in the presence of normal GLS but lower systolic blood pressure increased the likelihood of concurrent or subsequent cancer treatment–related cardiac dysfunction (CTRCD). Conversely, higher MWI reduced the risk of CTRCD when systolic blood pressure was elevated [65]. Kosmala *et al*. [66] found MW to be a more effective metric for assessing cardiotoxicity during follow-up in oncology patients undergoing chemotherapy, particularly when blood pressure varied by more than 20 mmHg. They discovered that global constructive work (GCW) and global myocardial work index (GWI) increased even when GLS decreased, indicating that increased afterload could affect left ventricular function without causing actual myocardial damage [66]. In summary, myocardial work serves as a more reliable indicator than GLS for monitoring chemotherapy-induced cardiotoxicity, particularly when accounting for blood pressure variations.

#### 2.5.3 Three-Dimensional Echocardiography

Three-dimensional echocardiography represents a significant advancement in ultrasound technology, offering real-time image acquisition from any spatial angle. This enhances the diagnostic capabilities of cardiac ultrasound, particularly for detecting cardiac tumors. Available in both transthoracic and transesophageal forms, 3D ultrasound has proven invaluable for evaluating heart structure, ventricular function, and valve architecture. Thavendiranathan *et al*. [67] compared 2D and 3D echocardiography to measure ejection fraction and volume in patients undergoing chemotherapy. Their findings indicate that 3D echocardiography offers more accurate and consistent assessments of ventricular function than its 2D counterpart [67]. Similarly, Khouri *et al*. [68] employed both 2D and 3D echocardiography to measure LVEF in asymptomatic early-stage breast cancer patients and controls. hey found 3D echocardiography to be more sensitive in detecting early subclinical cardiac dysfunction compared to 2D [68]. In summary, 3D echocardiography provides a more reliable method for calculating ventricular volume and function. It enhances the accuracy of LVEF and ventricular volume assessments, making it a superior tool for monitoring cardiac function in oncology patients. Given its advantages, 3D echocardiography should be more widely adopted to furnish clinicians with comprehensive and reliable data.

#### 2.5.4 Stress Echocardiography

Stress echocardiography (SE) is a well-established tool for evaluating ischemic cardiomyopathy. By increasing myocardial oxygen demand during a load test, SE helps to identify inadequate coronary blood flow and resultant myocardial ischemia, and comparing the echocardiographic performance of the resting and loading periods. Though commonly employed to detect coronary ischemia through exercise and dobutamine tests, SE is also useful for assessing ventricular dysfunction caused by radiotherapy-induced cardiotoxicity [69]. Ryerson *et al*. [70] showed the low sensitivity of conventional SE for detecting anthracycline-induced cardiotoxicity. Yet two-dimensional echocardiography (2DE) exercise stress echocardiography has shown utility in uncovering subclinical cardiac dysfunction that may not be apparent in resting 2DE, particularly following chemotherapy treatment for breast cancer [68]. This suggests that conventional SE is also useful in monitoring cardiotoxicity. In conclusion, there is a slight discrepancy between the value of conventional SE in monitoring cardiotoxicity. Thus, the combination of traditional SE and advanced ultrasound techniques can offer a more comprehensive approach to monitoring cardiotoxicity.

#### 2.5.5 Myocardial Ultrasonography

Conventional echocardiography has limitations in characterizing intracardiac masses, leading to a higher risk of misdiagnosis and errors. With the widespread use of acoustic contrast agents, myocardial echocardiography (MCE) has significantly improved the identification of cardiac tumors and occlusions [51]. One key advantage is its ability to differentiate vascular tumors from nonvascular tumors or thrombi based on varying levels of perfusion [51]. Microbubble contrast may invade vascular tumors, whereas thrombi or benign tumors usually have no increased perfusion [51]. MCE is divided into semi-quantitative and quantitative analysis methods. Semi-quantitative analysis involves visual observation: the occupying lesion is compared with the surrounding myocardium, malignant tumors are highly enhanced, benign tumors are overall low enhanced, positional lesions are dense thrombi, imaging lobing is obvious and no internal enhancement is sparse texture fresh thrombi [51]. MCE quantitative analysis was performed using correlation analysis software to calculate the contrast intensity-time curves A1/A2: a ratio greater than 1 was considered a malignant tumor, a ratio between 0 and1 was considered a benign tumor or thrombus, and a ratio equal to 0 was considered thrombus [51].

Combining the two approaches improves diagnostic accuracy. One study found that using MCE, when combined with qualitative and quantitative analyses, could accurately identify different types of cardiac masses [71]. Specifically, thrombi displayed no enhancement with an A1/A2 near 0, benign tumors had less enhancement than myocardium with an A1/A2 ratio between 0 and 1, and malignant tumors showed higher heterogeneous enhancements with an A1/A2 ratio above 1 [71]. Given these advancements, when a suspicious cardiac mass is detected through conventional 2D ultrasound and its nature remains uncertain, clinicians should employ further MCE imaging. This comprehensive approach enriches the clinical database and aids in more accurate diagnosis and management (Table 7, Ref. [60, 61, 62, 65, 66, 68, 70, 71]).

**Table 7.** **Major clinical studies of new ultrasound techniques to assess cardiac changes due to tumor patients**.

NO	First author	Year	New Technologies	Sample num	Main finding
1	Arciniegas Calle MC [60]	2018	2D-STE	66 patients with anthracycline-treated breast cancer	Early GLS changes predict chemotherapy-induced cardiotoxicity better than LVEF changes.
2	Coutinho Cruz M [61]	2020	Myocardial strain	105 patients with anthracycline-treated breast cancer	3D strain parameter prediction CTRCD outperforms 2D/3D LVEF and 2D GLS.
3	Luo R [62]	2017	Layered 2D-STE	44 patients with breast cancer treated with epi-adriamycin/controls group	Stratified strain technique enables early analysis of myocardial damage across all layers.
4	Ryerson AB [70]	2015	SE	80 cases of childhood cancer	Exercise echocardiography may not be valuable for routine monitoring of anthracycline cardiotoxicity.
5	Khouri MG [68]	2014	SE/3DE/GLS	57 patients with asymptomatic early breast cancer/control group	Resting 3DE, GLS, and exercise stress 2DE identify subcritical cardiac dysfunction undetectable in resting 2DE for breast cancer patients.
6	Li Y [71]	2022	Myocardial ultrasonography	108 patients with suspected cardiac occupancy by TTE	Myocardial angiography provides enhanced accuracy in identifying occupying lesions in the heart.
7	Calvillo-Argüelles O [65]	2022	MWI	136 HER2+ breast cancer patients treated with anthracyclines in combination with trastuzumab	In patients with a 3.3% change in GLS and a 21 mmHg decrease in systolic blood pressure, worsening GWI had a higher probability of merged CTRCD.
8	Kosmala W [66]	2022	MWI	44 asymptomatic patients at risk for CTRCD	Differences in the capacity of MW and GLS to detect reduced left ventricular systolic function induced by elevated blood pressure.

GLS, global longitudinal strain; LVEF, left ventricular ejection fraction; CTRCD, cancer treatment–related cardiac dysfunction; STE, speckle tracking echocardiography; SE, stress echocardiography; 2D, two-dimensional; 3D, three-dimensional; MW, myocardial work; 3DE, three-dimensional echocardiography; 2DE, two-dimensional echocardiography; TTE, transthoracic echocardiography; MWI, myocardial work index; HER2, human epidermal growth factor receptor 2; GWI, global myocardial work index.

## 3. Current Limitations

While echocardiography remains a valuable tool for the early diagnosis and evaluation of cardiac tumors, it’s important to acknowledge specific limitations. Factors including suboptimal image quality, particularly in the obese or patients with chronic lung disease, operator error, and the still undefined reference values for emerging technologies like 3D strain imaging can compromise diagnostic accuracy. Moreover, echocardiography may struggle to delineate the full extent and origin of cardiac masses located outside standard imaging fields (e.g., superior or inferior vena cava or pulmonary branch vessels). This constraint could lead to some degree of diagnostic error in the early detection of cardiac tumors and tumor-induced cardiovascular conditions. In addition, most studies have small sample sizes, brief follow-up periods, and lack of serum biomarker controls, which hinders drawing of robust conclusions. Therefore, while echocardiography provides substantial advantages in cardiac assessment, these constraints point to the need for further research and technological advancements to refine its diagnostic capabilities.

## 4. Conclusions

Echocardiography plays an important role in the early diagnosis, follow-up and management of cardiac issues in cancer patients. Advancements in ultrasound techniques, such as myocardial strain imaging, offer significant benefits in the assessment of cardiotoxicity. Specifically, the stratified myocardial strain technique, with its focus on a 10%–15% decrease in GLS shows promise as a key parameter. However, a standardized value for measuring myocardial longitudinal has yet to be established, warranting further investigation. One GLS limitation is the load dependency, which can be influenced by variations in blood pressure, even in the absence of changes in myocardial function, among patients undergoing oncology treatment. To address this limitation, MW can be adjusted for changes in GLS on systolic blood pressure, enhancing the reliability of GLS measurements. A baseline echocardiographic assessment should be conducted for most patients to facilitate early detection of cardiotoxicity, enabling timely cardiac intervention without interrupting critical oncologic treatment. Future research should focus on the impact of cardiotoxicity on right ventricular function, an underexplored area. In addition, emerging ultrasound techniques such as MCE continue to make breakthroughs in the identification of cardiac changes due to tumors and cardiac masses. Their application to cardiac lesions should be investigated in the future. Despite some limitations, echocardiography is a major cornerstone of cardiac oncologic monitoring because of its noninvasive, easy, real-time, and reproducible nature. As echocardiography continues to advance, it will play an increasingly crucial role in the early diagnosis, management, and prognosis of cardiac problems in oncology patients.
